# Recognition of Output-Side Series Arc Fault in Frequency Converter-Controlled Three-Phase Motor Circuit

**DOI:** 10.3390/s26030918

**Published:** 2026-01-31

**Authors:** Aixia Tang, Zhiyong Wang, Hongxin Gao, Congxin Han, Fengyi Guo

**Affiliations:** 1 College of Mining, Liaoning Technical University, Fuxin 123000, China; 471810020@stu.lntu.edu.cn; 2 Faculty of Electrical and Control Engineering, Liaoning Technical University, Huludao 125105, China; gaohongxin@lntu.edu.cn (H.G.); 471820204@stu.lntu.edu.cn (C.H.); 3 College of Electrical and Electronic Engineering, Wenzhou University, Wenzhou 325035, China; 20195207@wzu.edu.cn

**Keywords:** series arc fault, fault diagnosis, feature extraction, empirical wavelet transform, frequency converter-controlled three-phase motor circuit

## Abstract

Timely identification of series arc faults (SAFs) is of vital importance for preventing electrical fires. How to identify SAFs at the output side of a frequency converter (i.e., output-side SAF) is still not clear. A new approach of identifying output-side SAFs by analyzing the output current signals from frequency converters was proposed. First, output-side SAF experiments were performed with harmonic power supplies. Second, the output current signals were decomposed into eight modal components by empirical wavelet transform and an autoregressive model was established. The autoregressive model parameters and the energy ratios of the first three modal components were adopted as the fault features. Finally, an optimized support vector machine was designed and employed to identify SAFs. Comparison tests with similar methods were performed and performance tests under different noise levels and operation conditions were conducted. The test results indicated that the proposed scheme can effectively recognize output-side SAFs. Its runtime is shorter than 1.4 ms. This method provides a reference for the development of industrial three-phase SAF detection devices.

## 1. Introduction

In frequency converter-controlled three-phase motor (FCCTM) circuits, series arc faults (SAFs) are generated frequently, owing to poor electrical contacts on connection terminals or insulation deterioration of cables. SAFs can generate high temperatures of several thousand degrees during an arc duration. This can easily ignite combustibles near a fault spot within a very short time, resulting in electrical fires. Lots of studies have shown that conventional overcurrent protection equipment, such as molded case circuit interrupters and thermal relays, cannot identify such faults in time. Installing arc fault circuit interrupters (AFCIs) in circuits is an effective solution for implementing SAF protection. Currently, AFCIs suitable for industrial FCCTM circuits are uncommon and their fault recognition performance still cannot meet application requirements. Therefore, it is imperative to thoroughly explore the recognition principles for SAFs in FCCTM circuits. This is of great significance for the development of industrial three-phase AFCIs and the prevention of SAF-caused fire accidents.

In FCCTM circuits, the fault positions of SAFs are random. Therefore, it is difficult to identify SAFs by utilizing the physical features of arc discharge such as arc light [1], arc sound [2] and electromagnetic radiation [3]. Since it is convenient to collect circuit current signals in real time during engineering, SAFs in motor or FCCTM circuits are mainly identified by examining current signals. Qu [4] employed current amplitude and a sparse representation method to identify SAFs. Bao [5] and Chu [6] designed specialized current transformers and utilized high-frequency coupling current signals to recognize SAFs in household motor circuits. Artale [7] and Reji G. [8] analyzed the harmonic features of circuit current signals and applied the threshold method to detect SAFs. Zhou [9] and Di [10] utilized harmonic characteristics and several other indicators of current signals as fault features, and adopted a random forest algorithm and a stacking ensemble learning model, respectively, to recognize SAFs. Zhao [11] adopted multiple parameters of current signals to identify SAFs in angle grinder or vacuum cleaner circuits. Ji [12] applied current phase distribution characteristics within special frequency bands to identify SAFs in air conditioners or refrigerator circuits. Jiang [13] presented a signal decomposition and reconstruction solution to weaken the effect of a motor’s starting process on recognition accuracy. Calderon-Mendoza [14] used a Kalman filter and a fuzzy logic processor to identify SAFs in electric drill and air compressor circuits. Dai [15] converted current signals into a spectrogram and utilized a residual network to detect SAFs. Hua [16] extracted fault features from Fast Fourier Transform spectra of current signals, then adopted a Newton-optimized sparse kernel support vector machine (SVM) to identify SAFs. Park [17] examined the performance of several neural network-based SAF recognition methods. Zhang [18] proposed an adaptive asymmetric convolutional neural network (CNN)-based SAF recognition model. Wang [19] deployed a recognition model named ArcNet to a hardware platform to identify SAFs in a household load circuit. Saleh [20] presented an approach to identify SAFs in three-phase motor circuits. Shen [21] calculated the non-periodic fault features of SAFs in a squirrel cage motor circuit. Yu [22] detected three-phase SAFs using an improved EfficientNetV2 algorithm. Gao [23] adopted fractional Fourier transform with two-level singular value decomposition (SVD) to obtain arc fault features in an FCCTM circuit. Han [24] and Liu [25] designed recognition models of SAFs in FCCTM circuits by considering the influences of harmonic sources and motor vibration conditions, respectively. Wang [26,27] proposed a recognition approach for two-phase SAFs in an FCCTM circuit and further developed an on-line SAF detection device for the circuit based on a lightweight recognition model.

In summary, the existing SAF recognition methods suitable for motor or FCCTM circuits still have the following deficiencies:

(1) The method in [4] is suitable for DC motor circuits, while the methods in [5,6,7,8,9,10,11,12,13,14,15,16,17,18,19] are appropriate for domestic single-phase motor circuits. As we all know, several aspects of industrial three-phase motor circuits differ from those of family single-phase motor or DC motor circuits. These aspects include power supply and its power quality, motor type and its working characteristics, service environment and so on. Such differences make the above-mentioned methods unable to accurately recognize SAFs in three-phase motor circuits.
(2) Although the methods in [20,21,22] can recognize SAFs in three-phase motor circuits, the effect of frequency converters on fault recognition performance has not been considered. The existence of frequency converters makes identification of SAFs more complicated. There are two types of SAFs in FCCTM circuits. One is input-side SAFs, i.e., SAFs occur at the input side of a frequency converter. The other is output-side SAFs. It means SAFs occur at the output side of a frequency converter. The power electronics circuits inside a frequency converter make its output current signals contain lots of harmonics and burrs. They will mask or weaken fault features contained in current signals, making it very challenging to identify output-side SAFs. Although the methods in [23,24,25,26,27] can identify input-side SAFs in FCCTM circuits, whether they can still accurately identify output-side SAFs has not been verified.

**Figure 1:**
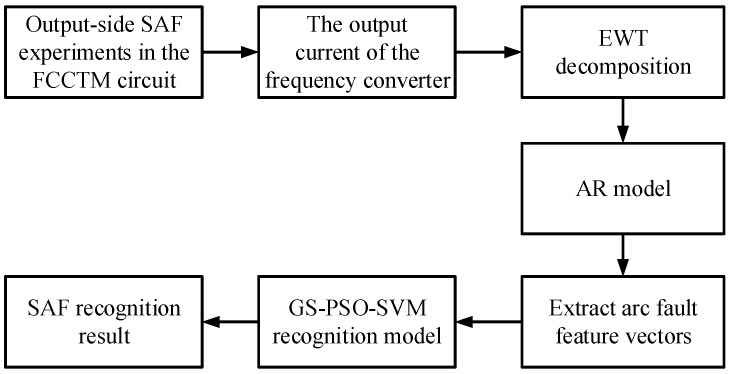
The flowchart of the overall work.

To solve these problems, research on recognition of output-side SAFs in FCCTM circuits was conducted. The flowchart of the overall work is shown in Figure 1. Output-side SAF experiments were first conducted in a FCCTM circuit under complex harmonic conditions. Then, empirical wavelet transform (EWT) and autoregressive (AR) model were introduced to analyze the output current signal from a frequency converter to extract fault features. And a well-trained SVM was used to identify output-side SAFs. Finally, effectiveness of this method was inspected by numerous tests.

The main innovations and academic contributions of this work are as follows:

(1) A new idea of identifying output-side SAFs by analyzing output current signals from frequency converter was proposed for the first time. It not only broadens research field of SAFs in FCCTM circuits, but also provides a new approach for SAF detection in industrial three-phase motor circuits.
(2) A novel feature extraction method of output-side SAFs in FCCTM circuits was proposed based on EWT and AR model parameters. It comprehensively considers the anti-interference performance, adaptability to working conditions and convenience of hardware deployment. Combined with an SVM, the simple and feasible approach can effectively recognize output-side SAFs in FCCTM circuits. It provides a reference for the development of industrial three-phase AFCIs.

The rest of this article is organized as follows. Section 2 introduces SAF experiments and analyzes measured current signals. Section 3 describes the principle of EWT and the extraction method of fault features. Section 4 presents the proposed SAF identification method. It also discusses the effectiveness of this method under different conditions. Section 5 presents the conclusions.

## 2. SAF Experiments

### 2.1. Experimental Scheme

**Figure 2:**
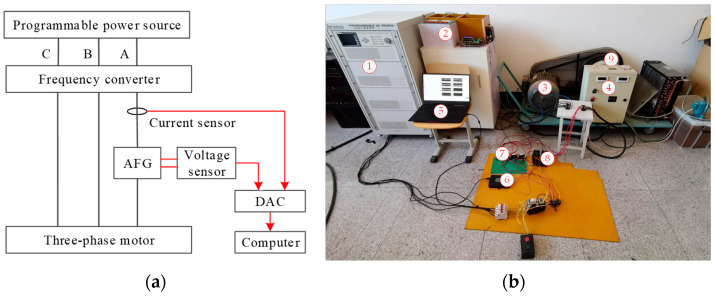
The experimental platform. (**a**) Principle diagram; (**b**) photo. ① programmable power source; ② AFG; ③ three-phase motor; ④ frequency converter; ⑤ computer; ⑥ DAC; ⑦ current sensor; ⑧ voltage sensor; ⑨ friction load.

The experimental platform in Figure 2 was used to perform SAF experiments. A Chroma 6590 type programmable power source (Chroma ATE Inc., Taiwan, China) was used to generate three-phase output voltage with various harmonics. Its rated output voltage and output frequency are AC 380 V and 50 Hz, respectively. Its maximum output current is 20 A. A VFD110E43A type frequency converter (Delta Greentech (China) Co., Ltd., Shanghai, China) and an Y160M-6 type three-phase asynchronous motor (Hengshui Funiu Electric Machinery Co., Ltd., Hengshui, China) were connected in series and used as experimental load. The frequency converter is a vector model one, and it adopts an open-loop vector control method. Its rated input voltage is 380 V with a frequency of 50 Hz. Its rated power is 1.5 kW. Its output frequency range is from 0 to 450 Hz, and carrier frequency range is from 1 to 15 kHz. The three-phase motor is a six-pole motor, connected in delta configuration. Its rated power is 11 kW, rated speed is 970 r/min and power factor is 0.78. The motor is coaxially connected to an FZYJ400 type friction load (Wuxi YUKEN Technology Co., Ltd., Wuxi, China). The working current of the three-phase motor can be regulated between 12 A and 20 A by adjusting the operation state of the friction load. An arc fault generator (AFG) was developed according to UL 1699 standard [28]. The AFG was connected to A-phase circuit between the three-phase motor and the frequency converter. It was used to produce output-side SAFs equivalent to the actual ones. The structure and working principle of AFG, as well as specific operation steps of generating SAFs, were described in detail in [29].

An LHB 100A5VY2 type hall current sensor (Zhuhai Starlight Electronics Technology Co., Ltd., Zhuhai, China) was adopted to detect A-phase output current signal of the frequency converter. Its frequency bandwidth is from DC to 150 kHz and measurement accuracy is 1%. An LHB 500V5VT type hall voltage sensor was used to detect the voltage signal across the static and movable electrodes of the AFG. Once SAFs occur, the voltage is arc voltage. An arc voltage signal is only used to judge circuit working state, not to identify SAFs. A USB-3200 type 12-bit data acquisition card (DAC) (Bejing Art Technology Development Co., Ltd., Beijing, China) was used to transmit the current and voltage signals to a computer for further analysis. The sampling frequency was set to 10 kHz.

As shown in Table 1, six three-phase power sources labeled as U1–U6 were used to conduct the experiments. Among them, U1 is a commercial power source with a rated frequency of 50 Hz. U2–U6 are obtained from the programmable power source by setting different parameters. U2 outputs ideal sinusoidal voltage, whose amplitude and frequency are 380 V and 50 Hz, respectively. U3–U6 are power sources with harmonics noise. The corresponding harmonic contents and phase angle are shown in Table 2 [30]. For each power source, the experiments were performed both in normal and fault state. That is, there is no SAF in the circuit, or there are output-side SAFs generated by the AFG. Therefore, a total of 12 groups of experiments were conducted. For all experiments, current is 12 A, and the working frequency and PWM carrier frequency of the frequency converter are 50 Hz and 8 kHz, respectively.

### 2.2. Experimental Results and Analysis

**Figure 3:**
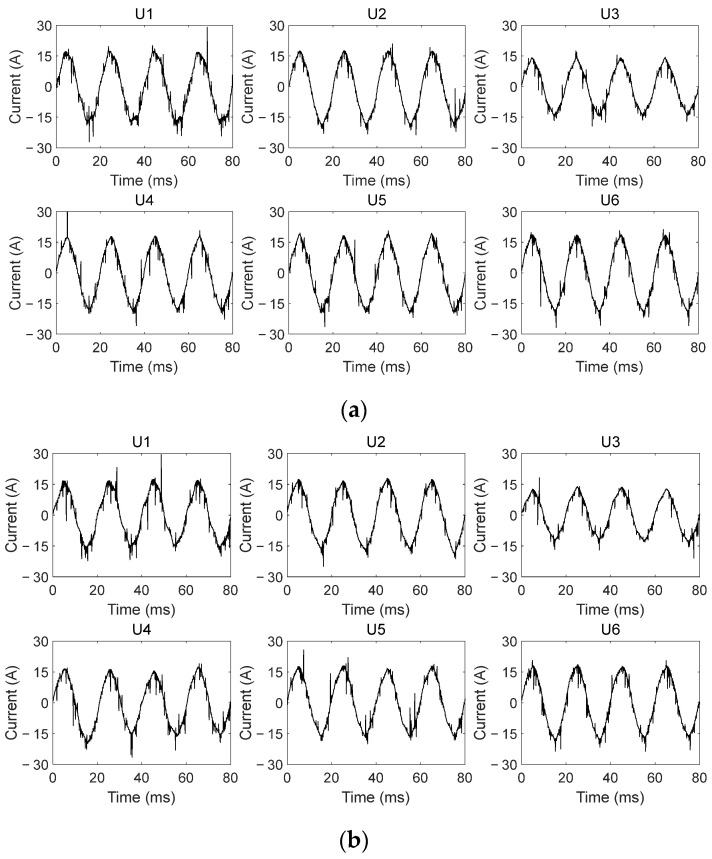
Typical output current waveforms of the frequency converter in the FCCTM circuit: (**a**) in normal state; (**b**) in fault state (i.e., output-side SAFs occur).

**Figure 4:**
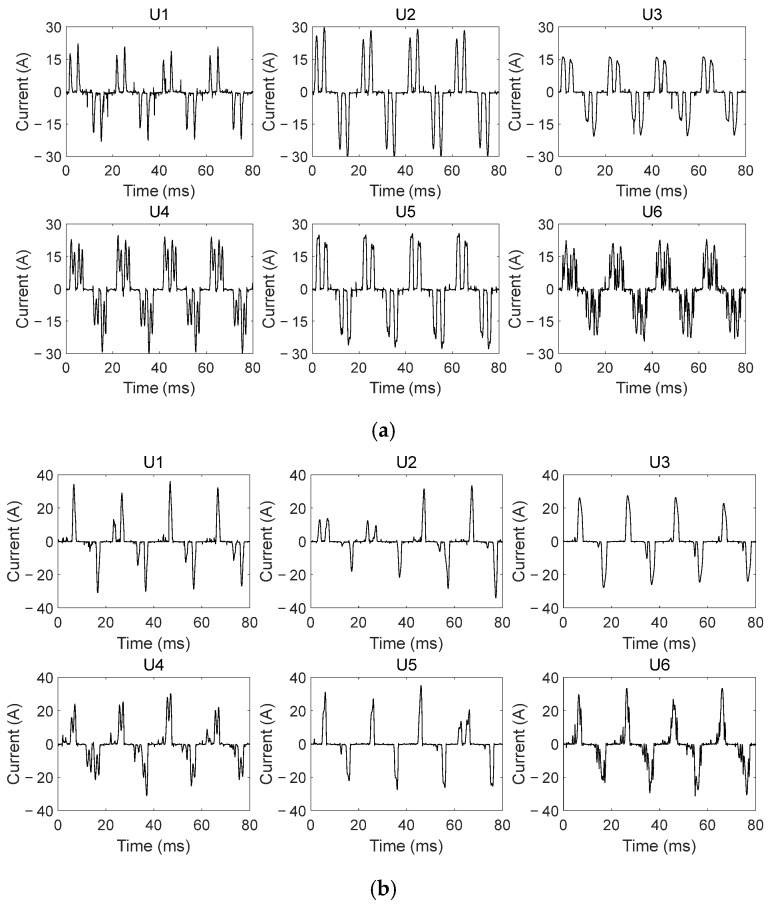
Typical input current waveforms of the frequency converter in the FCCTM circuit [30]: (**a**) in normal state; (**b**) in fault state (i.e., input-side SAFs occur).

Figure 3 and Figure 4 are typical output and input current waveforms of the frequency converter, that is, the currents were detected at the output side and input side of the frequency converter, respectively.

As seen in Figure 3 and Figure 4, there are more spikes in output current signals under the same experimental conditions. In a normal state, even if the ideal power source is used, there are still lots of spikes in output current signals. These spikes are generated by power electronics devices inside the frequency converter. It means that the frequency converter varies the characteristics of output current signals. Moreover, even in the same working state, power supplies with different harmonic components cause certain differences in the number and amplitude of the spikes in output current signals. It demonstrates that the power quality further affects output current signals, which makes the current characteristics more complicated.

Compared with those in a normal state, when output-side SAFs occur, output current waveforms do not change significantly. Only the amplitudes and quantities of the spikes in waveforms vary to different degrees. However, due to the effect of the frequency converter and of the burning state of SAFs, the quantities of the spikes in fault current waveforms may increase and may also decrease. Furthermore, the waveform characteristics of different current cycles are also different. There is no specific pattern in changes of these spikes. These weak arc fault features will be submerged to some extent by existing spikes in the current waveforms. It brings more challenge to recognizing output-side SAFs. So, it is necessary to effectively mine fault features from the output current signal by utilizing appropriate feature extraction methods. It is very crucial for accurate recognition of output-side SAFs in FCCTM circuits.

## 3. Extraction of Fault Features

To facilitate extraction of weak arc fault features from complex output current signals, the current signal was first decomposed into multiple signal components, and then the fault feature vector of output-side SAFs was constructed by using the AR model.

In the SAF detection field, methods such as empirical mode decomposition (EMD), wavelet transform (WT), wavelet packet transform (WPT) and variational mode decomposition (VMD) are adopted to decompose current signals. However, EMD has problems such as mode mixing and end effects. Discrete WT and WPT algorithms utilize a specific frequency band to decompose the signals. When the true frequencies of the signal components are different from the pre-determined frequency band, such methods cannot accurately separate these signal components. VMD has a problem of large computation. EWT is a signal decomposition method based on wavelet analysis. It has advantages of no mode mixing and small computation. Therefore, EWT and AR model were combined to extract fault features of SAFs from the output current.

### 3.1. EWT of the Current Signal

EWT can adaptively decompose a complex non-stationary signal into several modal components with specific frequency characteristics and time-frequency local properties via orthogonal wavelet filters [30,31]. The number of decomposition layers of the signal needs to be determined first before performing EWT. The number of decomposition layers (labeled as *K*) will directly affect the resolution of decomposition results and the degree of modal aliasing. If *K* is too small, it may cause modal aliasing, thereby affecting the accuracy of subsequent analysis. Otherwise, if *K* is too large, it may lead to over-decomposition, resulting in a large number of invalid or noise components and increasing computational burden. In this work, *K* was determined based on EMD decomposition results of a large amount of output current signals. Its rationality was verified in Section 4.3.1 by comparing identification accuracies of SAFs under different *K*-value conditions. EMD decomposes a signal in light of time-scale features of the signal itself. It satisfies residual requirements by calculating envelopes and time series many times, and can reflect the number of modes contained in the original signal [32]. So the number of decomposition layers of EWT on a signal can be determined by EMD.

For each experiment in Table 1, 100 samples were truncated from the measured current signal. The length of each sample is four current cycles. Since current frequency is 50 Hz and sampling frequency is 10 kHz, each sample contains 800 data points. EMD was performed on these signals. By analyzing the obtained EMD results, it is found that the number of decomposition layers of these current signals is between 6 and 8. So the number of decomposition layers was set to 8.

**Figure 5:**
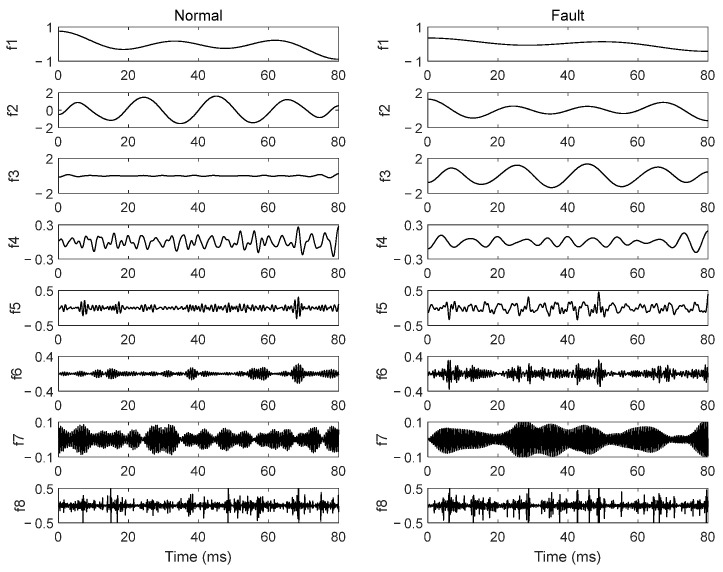
EWT results of the current signal under U1 conditions.

To reduce the effect of current amplitude on feature extraction, the output current signal was first normalized via root mean square. Then it was decomposed into 8 modal components by EWT. They are respectively denoted as f1–f8. Taking the current signal under U1 conditions as an example, the EWT results are demonstrated in Figure 5. As seen in Figure 5, when output-side SAFs occur, all the eight modal components have some changes. The fault features in these modal components are more obvious than those in the original current signal.

The energy ratios of these modal components were calculated by Equation (1), as shown in Table 3.

(1) $$r_{i}=\frac{E_{i}}{E}=\frac{{\sum_{1}^{N}\left|x_{i}(n)\right|}^{2}}{{\sum_{1}^{N}\left|x(n)\right|}^{2}}$$

where *E* is the total energy of a signal. *E_i_* is the energy of the *i*-th modal component. *r_i_* is the energy ratio of the *i*-th modal component, *i* = 1, 2… *K*. *K* is the total number of modal components. In this work, *K* = 8. *x_i_*(*n*) is the value of the *i*-th modal component at discrete time point *n*. *x*(*n*) is the value of the signal at discrete time point *n*. *N* is the total number of sampling points of the signal.

As indicated in Figure 5 and Table 3, the modal components f1–f3 contain the main energy of the current signal and their waveforms are relatively simple, which is suitable for solving model parameters of an AR model. Furthermore, when output-side SAFs occur, the energy ratios of these three modal components change obviously. Therefore, the first three modal components of EWT results were chosen to calculate fault features of SAFs.

### 3.2. Fault Features of SAFs

The AR model is a commonly used time series analysis method. Its core principle is to predict current value through a linear combination of historical observation values. Its mathematical expression is

(2) $$X_{t}=c+\phi_{1}X_{t-1}+\phi_{2}X_{t-2}+\cdots +\phi_{P}X_{P-1}+\varepsilon_{t}$$

where *X_t_* is the value of the time series at time *t*. *c* is a constant term. *ϕ*_1_, *ϕ*_2_… *ϕ_P_* are model parameters, namely autoregressive coefficients. *P* is model order. *ε_t_* is a random error term. It is usually assumed to follow random white noise with a mean of 0 and a variance of σ^2^.

The model order *P* has a significant impact on analysis results. An excessively high model order leads to an overly complex model, while an excessively low model order fails to capture dynamic characteristics of the time series. After analyzing experimental data, based on Akaike’s final prediction error criterion [33], *P* was set to 3. The third-order Burg algorithm was used to calculate AR model parameters.

AR model parameters and energy ratios of the first three modal components of EWT results were employed to create a feature vector of SAFs. The detailed steps are as follows:

(1) Collect a four-cycle current signal from output current signals of the frequency converter, and normalize it by root mean square.
(2) Decompose the normalized signal into eight modal components by EWT.
(3) Construct an AR model with the first three modal components by using third-order Burg algorithm.
(4) Calculate AR model parameters and energy ratios of the first three modal components, normalize and then take them as the fault features.

**Figure 6:**
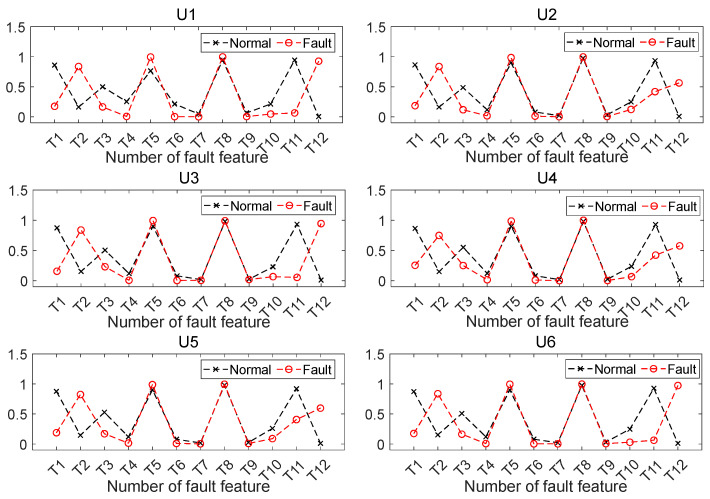
Extracted fault features.

The fault features under different power source conditions are demonstrated in Figure 6. In Figure 6, T1–T3 are three model parameters of the third-order AR model of the first modal component, respectively. Correspondingly, T4–T6, T7–T9 are these three parameters of the second and third modal components, respectively. T10–T12 are energy ratios of the 1st, 2nd and 3rd modal components, respectively. Figure 6 shows that several fault features present different degrees of variations when SAFs occur. It indicates that SAFs can be identified theoretically by using these fault features.

## 4. SAF Recognition and Performance Analysis

### 4.1. Recognition Method

In recent years, SVM has been widely applied in the fault diagnosis field. SVM has superior performance in handling small sample sizes, high-dimensional data, and nonlinear problems. To further improve the classification ability of SVM, extensive research in various aspects, such as construction of kernel functions, parameter optimization and model structure improvement, has been conducted. For example, Akbari [34] proposed a classification method combining a semi-parametric model with SVM. By introducing a dual kernel mechanism, it integrates linear interpretability with nonlinear modeling capability, thereby improving classification performance for complex boundary data. In addition, different intelligent optimization algorithms, such as particle swarm optimization, genetic algorithm and firefly optimization algorithm, have been applied to optimize model parameters of SVM.

To examine the availability of the proposed fault feature extraction scheme, an SVM with radial basis function kernel function was applied as a classifier to identify output-side SAFs. In this classifier, penalty factor *c* and kernel function parameter *g* are crucial to classification performance. They affect the generalization performance and computational complexity of the classifier. Parameters *c* and *g* are usually determined by methods such as the trial-and-error method, empirical setting method, grid search (GS) algorithm, and heuristic optimization algorithms. Typical heuristic optimization algorithms include particle swarm optimization (PSO), genetic algorithm, ant colony optimization algorithm, simulated annealing algorithm and so on. In this work, grid search and particle swarm optimization (GS-PSO) algorithm [23] was adopted to determine optimal parameters *c* and *g*.

GS-PSO algorithm has advantages of both GS algorithm and PSO algorithm. It can enhance computational efficiency and reduce computational cost on the basis of ensuring the optimization effect. The overall idea of the GS-PSO algorithm is as follows. First, the PSO algorithm is used to obtain optimal parameters. It is performed within a wide parameter search range and with a large search step size. Then, a narrower parameter search range is set around the obtained optimal parameters, and a smaller search step size is set. Finally, the GS algorithm is used to find the optimal parameters.

The specific steps of optimizing parameters *c* and *g* via the GS-PSO algorithm are as follows. (a) Set search ranges for parameters *c* and *g* to [*c*_1*min*_, *c*_1*max*_] and [*g*_1*min*_, *g*_1*max*_]. (b) Determine initial parameters of the PSO algorithm. The main initial parameters include the maximum number of iterations *gen_max_*, population size *popsize* and so on. (c) Perform PSO algorithm to obtain optimal parameters *c* and *g*, labeled as *c_opt_*_1_ and *g_opt_*_1_, respectively. (d) Determine search intervals [*c*_2*min*_, *c*_2*max*_] and [*g*_2*min*_, *g*_2*max*_] for parameters *c* and *g* in the GS algorithm, and set smaller search step sizes *d_c_*_2_ and *d_g_*_2_ of these two parameters. (e) Calculate recognition accuracy of each grid node in the GS search grid. It is achieved by substituting parameters *c* and *g* of the grid node into the SVM classifier. (f) Find the node with the highest recognition accuracy, and determine the parameters *c* and *g* of this node as optimal parameters *c_opt_* and *g_opt_*.

**Figure 7:**
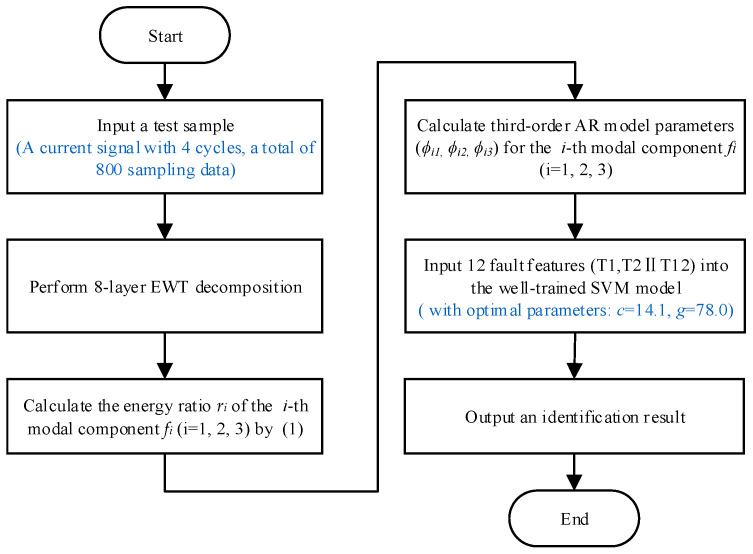
The flowchart of the proposed SAF identification method.

The flowchart of the proposed SAF identification method is shown in Figure 7.

### 4.2. Model Training and Test

#### 4.2.1. Dataset Creation

For each experiment in Table 1, 200 samples were truncated from measured current signals in normal and fault state. The length of each sample is four current cycles. Each sample has 800 data points. Using the method in Section 3.2, fault features were extracted from the current waveform of each sample. And category labels of the obtained fault feature vectors were defined. The fault feature vectors corresponding to the current waveforms in normal and fault state are defined as normal samples and fault samples, respectively. The category label of normal samples is 1, while that of fault samples is 0. In this way, a sample dataset under six different harmonic power supply conditions was obtained and labeled as DATASET_A. The dataset consists of 1200 normal samples and 1200 fault samples. It was evenly divided into a training set and a test set. The ratio of normal samples and fault samples in the training set and the test set is all 1:1.

#### 4.2.2. Optimization of SVM Parameters and Model Training

The optimal SVM parameters *c* and *g* can be obtained by performing the GS-PSO algorithm. The main initial parameters of the algorithm were set as follows. The parameters *c*_1*min*_ and *c*_1*max*_ were set to 0.001 and 1000, respectively. The parameters *g*_1*min*_ and *g*_1*max*_ were also set to 0.001 and 1000, respectively. The maximum number of iterations *gen_max_* was set to 100. The population size *popsize* was set to 20. The optimal parameters *c* and *g* obtained from the PSO algorithm were labeled as *c_opt_*_1_ and *g_opt_*_1_, respectively. The search interval of parameter *c* in the GS algorithm was $\left[2^{\log_{2}c_{opt1}-1},2^{\log_{2}c_{opt1}+1}\right]$, and that of parameter *g* was $\left[2^{\log_{2}g_{opt1}-1},2^{\log_{2}g_{opt1}+1}\right]$. The search step size of parameter *c* in the GS algorithm was $2^{0.05(\log_{2}c_{opt1}-1)}$, and that of parameter *g* was $2^{0.05(\log_{2}g_{opt1}-1)}$. Based on these parameters and one quarter of training samples, optimal parameters *c* and *g* were obtained by performing the GS-PSO algorithm. They are 14.1 and 78.0, respectively.

The SVM was trained with training samples in DATASET_A. The well-trained SVM was utilized to recognize SAFs.

#### 4.2.3. Recognition Performance Test

Six indicators were adopted to comprehensively evaluate the recognition performance of the identification method. They are accuracy, precision, recall, F1-score, false positive rate (FPR) and false negative rate (FNR), respectively. They can all be calculated based on the elements in the confusion matrix of each test result.

**Figure 8:**
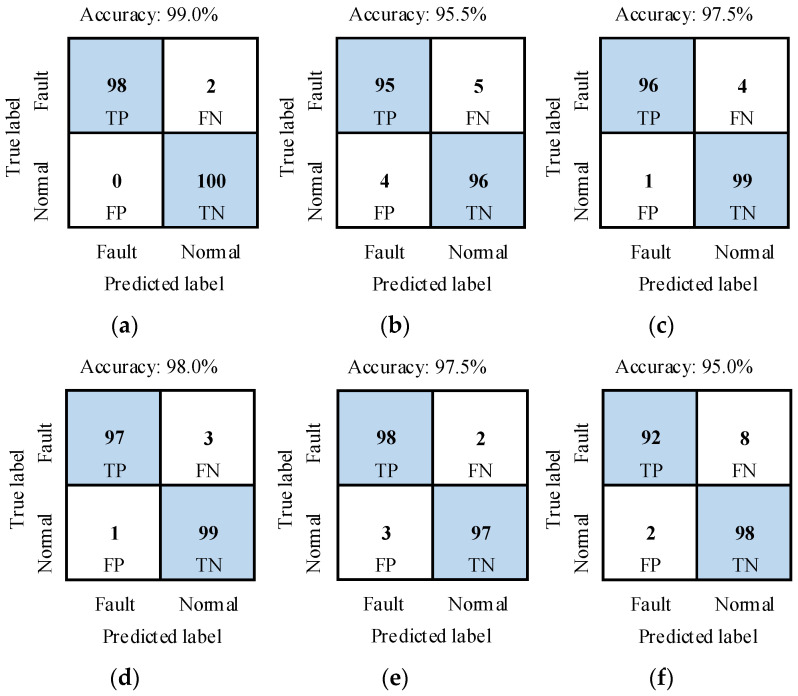
Confusion matrices of recognition results under different power source conditions. (**a**) U1; (**b**) U2; (**c**) U3; (**d**) U4; (**e**) U5; (**f**) U6.

The recognition performance of the method was tested with test samples in DATASET_A. The recognition results of SAFs under different power source conditions are shown in Figure 8 and Table 4. In Figure 8, TP, FN, FP and TN are four elements of a confusion matrix. They are true positive, false negative, false positive and true negative, respectively. The results showed that average accuracy is 97.1%, and average false detection rate (i.e., FPR) and average missed detection rate (i.e., FNR) are 1.8% and 4%, respectively. So the recognition performance of the proposed method is satisfied.

### 4.3. Rationality Tests on Key Parameters of the Proposed Method

#### 4.3.1. Selection on Number of EWT Decomposition Layers and AR Model Order

In Section 3.1, eight-layer EWT decomposition was conducted and the first three modal components were used as feature components. To verify its rationality, another 200 samples were truncated from measured current signals in normal and fault state of each power source in Table 1. Fault features were constructed under the conditions of different numbers of the decomposition layers (labeled as *K*) and different numbers of the modal components (labeled as *M*). All the samples were evenly divided into a training set and a test set. The ratio of normal samples and fault samples in the training set and the test set is all 1:1. For each combination of parameters *K* and *M*, parameters *c* and *g* of the SVM were re-optimized and the model was re-trained by using training set samples. Recognition tests were performed by inputting test set samples into the well-trained recognition model. Test results are shown in Table 5. In Table 5, recognition accuracy is the highest when *K* is 8 and *M* is 3. So it is reasonable to decompose current signals into 8 layers and use the first 3 modal components to create fault features.

**Figure 9:**
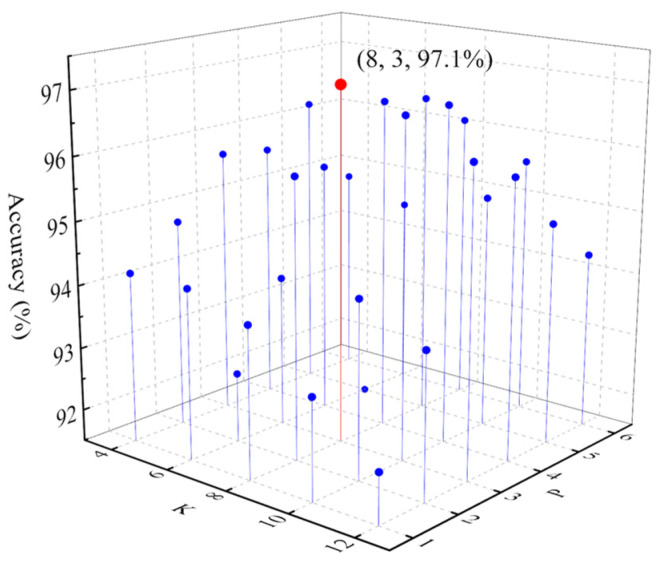
Recognition accuracy under different *K* and *P* conditions.

To further verify the rationality of the selection of AR model order, the recognition accuracies under the conditions of different numbers of the decomposition layers (labeled as *K*) and different AR model order (labeled as *P*) were tested with the above-mentioned method. *K* increases from 4 to 12, with a step size of 2. *P* increases from 1 to 6, with a step size of 1. When the first three modal components of EWT decomposition results were used to extract fault features, the identification results are shown in Figure 9. It shows that recognition accuracy is the highest when *K* is 8 and *P* is 3. Therefore, it is reasonable to decompose current signals into 8 layers and use third-order AR model parameters to create fault features.

#### 4.3.2. Selection on Sampling Frequency and Sample Length

Sample length will change the number of data sampling points in each sample. Sampling frequency will affect sampling accuracy of a signal. Therefore, in essence, sample length and sampling frequency will influence signal quality of sample signals, and thereby affect fault features of the signals and recognition performance of the recognition method. To verify rationality of sample length and signal sampling frequency in the proposed method, recognition performance tests under different sample length and signal sampling frequency conditions were conducted. The test scheme is shown in Table 6.

Tests were conducted by using the experimental data in Table 1. In the experiments in Table 1, sampling frequency of the current signal was 10 kHz. The frequency sampling method was adopted to obtain current signals with the sampling frequency of 5 kHz and 2.5 kHz from measured current signals. For each test, 200 samples were truncated from current signals in normal and fault state of each power source. Fault features were extracted with the method in Section 3. All samples were evenly divided into a training set and a test set. The ratio of normal samples and fault samples in the training set and the test set is all 1:1. The recognition model was trained with training set samples by using the method in Section 4.1. Then, recognition performance was tested by inputting test set samples into the well-trained recognition model. Test results are shown in Table 6.

As seen in Table 6, under the same signal sampling frequency (i.e., 10 kHz) conditions, recognition accuracy is the best when the sample length of each sample is 4 current cycles. Although such recognition performance could also be achieved when sample length is 7 current cycles (i.e., the B17-th group test), the number of sampling points for each sample increases from 800 to 1400. This would to some extent increase computational complexity of feature extraction. Therefore, sample length was set to 4 current cycles. When sample length is 4 current cycles, the recognition performance declines with the continuous decrease of signal sampling frequency. When sampling frequency is 10 kHz, recognition accuracy is 97.1% and F1-Score is 0.97. It indicates that the proposed method can effectively identify SAFs. Therefore, the selection of both signal sampling frequency and sample length is reasonable and effective.

### 4.4. Comparison with Existing Methods

#### 4.4.1. Comparison with Different Signal Decomposition Methods

To evaluate the superiority of EWT in signal decomposition of output current signals, recognition performance tests with different signal decomposition methods were conducted.

For each experiment in Table 1, 200 normal samples and 200 fault samples were randomly selected. So the dataset (labeled as DATASET_CA) contains 1200 normal samples and 1200 fault samples. The length of each sample is four current cycles. The current signals of each sample were decomposed with four signal decomposition methods. These methods are EMD, WPT, VMD and EWT, respectively. Optimal decomposition results were adopted for each method. EMD automatically decomposes these signals into 6 to 8 layers, while WPT, VMD and EWT all decompose these signals into 8 layers. For each method, the first three modal components of the decomposition results were used to construct a three-order AR model. Fault features were extracted with the method in Section 3.2. The dataset was divided into a training set and a test set with the method in Section 4.1. And the recognition model was trained and tested with the method in Section 4.2. In addition, to analyze the computation of different signal decomposition methods, average runtime per signal decomposition was calculated. The program code of the recognition method was written and run in Matlab v2023b software. The software was installed on a computer. The CPU of the computer is Intel(R) Core(TM) i5-9500. Its main frequency is 3 GHz. The memory of the computer is 8 GB. The operating system of the computer is a 64-bit Windows 10 system. The test results are displayed in Table 7.

Table 7 shows that the recognition performance of EWT is much higher than that of EMD, WPT, and VMD. And the average runtime of EWT is the shortest. It means EWT is better than the other three signal decomposition methods.

#### 4.4.2. Comparison with Different Feature Extraction Methods

To examine the superiority of the presented feature extraction method, comparison tests with the following eight methods were carried out.

Method 1 [35]: The Db10 wavelet basis function was utilized. The current signal was decomposed into 3 layers. The energy of high-frequency components was calculated and employed as SAF features.

Method 2 [7]: The signal difference between the low-frequency spectrum signals of two sections of current was first calculated. Then, the mean and the two-order, four-order and six-order harmonic change rates of the signal difference were extracted and adopted as feature variables.

Method 3 [23]: First, the current signal was de-noised by using the wavelet threshold algorithm and a difference calculation was conducted. Then, fractional Fourier transform and a two-block SVD was performed on the signal. Finally, the first 30 singular values were calculated and utilized as SAF features.

Method 4 [30]: The current signal was decomposed into five empirical modal functions (EMFs) using EWT. Attractor matrices were constructed by using these EMFs, and the first two singular values of each attractor matrix were extracted and used as fault features.

Method 5 [36]: The kurtosis, crest factor, clearance factor, L2/L1 norm, shape factor and spectral centroid were extracted from the current signal and used as fault features.

Method 6 [37]: The mean current value, harmonic amplitudes and wavelet energy entropy were calculated from the current signal and adopted as fault features.

Method 7 [38]: The current signal was decomposed using EWT. Ten time-domain features and four entropy-based features were extracted from the sixth EMF. The time-domain features are variance, mean, kurtosis, margin factor, waveform factor, kurtosis factor, impulse factor, root mean square, skewness and rectification average value, respectively. The entropy-based features are sample entropy, fuzzy entropy, permutation entropy and approximate entropy, respectively. The principal components with a cumulative contribution rate higher than 90% were selected by principal component analysis to reduce the dimensions of the features.

Method 8: The current signal was decomposed into eight layers using EWT. The absolute average value, standard deviation, skewness and kurtosis of the first three modal components of decomposition results were calculated. The obtained twelve time-domain indicators were used as fault features.

Performance tests of these eight methods were conducted on the same dataset, which is labeled as DATASET_CB. Its creation method is similar to that of DATASET_CA. It also consists of 1200 normal samples and 1200 fault samples. They are randomly selected from measured current signals under the conditions of six power supplies. The length of each sample is four current cycles. Fault features were extracted with the eight methods in Table 8. The dataset was divided into a training set and a test set with the method in Section 4.1. And the recognition model was trained and tested with the method in Section 4.2. The test results are displayed in Table 8.

In Table 8, the recognition accuracy of methods 1, 3, 5 and 6 is much lower than 85%. This is because the output current signal of the frequency converter is very complex and contains rich high-frequency harmonics even if there is no SAF in the circuit. Therefore, low-frequency features of the output current signal should be used to identify output-side SAFs. Therefore, these four methods cannot accurately identify SAFs. Although the runtime of methods 2, 4, and 5 is shorter than that of the proposed one, its recognition accuracy is much poorer. Although the recognition accuracy of methods 7 and 8 is higher than 90%, it is still lower than that of the proposed method. Moreover, the runtime of the 7th method is much longer, which cannot meet real-time requirement of SAF detection. So, the proposed method has the best recognition performance among these methods.

### 4.5. Anti-Interference Performance Tests

To verify anti-interference performance of the proposed recognition method, recognition performance tests were conducted under two kinds of noise and three kinds of noise intensity. Gaussian white noise and pink noise were used in this work. The signal-to-noise ratio (SNR) was used to reflect noise intensity. A lower SNR indicates a higher intensity of noise in the signal. The noise intensities of Gaussian white noise are 30 dB, 25 dB and 20 dB, respectively. The noise intensities of pink noise are 40 dB, 35 dB and 30 dB, respectively.

**Figure 10:**
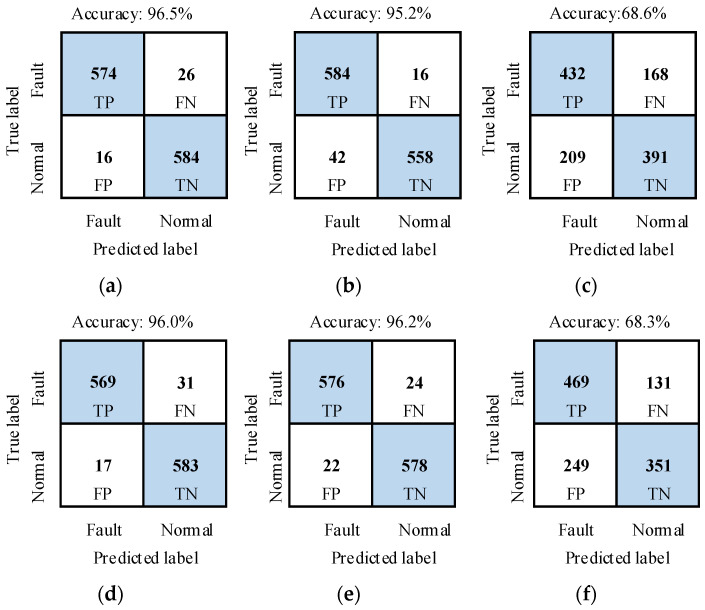
Confusion matrices of identification results of test groups D11–D13 and D21–D23. (**a**) D11; (**b**) D12; (**c**) D13; (**d**) D21; (**e**) D22; (**f**) D23.

For each experiment in Table 1, 100 samples were truncated from measured current signals in normal and fault states. So, there are 600 normal samples and 600 fault samples in the dataset. The length of each sample is four current cycles. The dataset was labeled as DATASET_D. All the samples in the dataset were only used as test samples. The Gaussian white noise with three kinds of noise intensity and that of pink noise were added to the current signal of each sample. Fault features were extracted from sample current signals with interference noise by using the method in Section 3.2. The well-trained recognition model in Section 4.2 was employed to conduct performance tests. The test results are presented in Figure 10, and Table 9 and Table 10. When the SNR of Gaussian white noise is not less than 25 dB or that of pink noise is not less than 35 dB, the recognition accuracy of the method is higher than 95%. It indicates that the proposed method has certain anti-noise interference capability.

### 4.6. Effectiveness Tests Under Different Conditions

#### 4.6.1. Effectiveness Tests Under Different Circuit Operation Parameter Conditions

To evaluate the effectiveness of the proposed method under different circuit operation parameter conditions, additional SAF experiments were conducted by using the experimental platform in Figure 2. The commercial power supply was used in the experiments. Its rated output voltage is 380 V and its rated frequency is 50 Hz. Seven groups of experiments were carried out by changing working current, as well as working frequency and PWM carrier frequency of the frequency converter. The experimental conditions are shown in Table 11. To assess the impact of operation parameter fluctuations on recognition performance, an SAF experiment under fluctuating working current conditions was conducted. It is the E7-th experiment in Table 11. The working current fluctuated randomly between 12 A and 16 A. A-phase output current signal of the frequency converter was collected in real-time. The sampling frequency is 10 kHz.

For each experiment in Table 11, 100 samples were truncated from measured current signals in normal and fault states. So, there are 700 normal samples and 700 fault samples in the dataset. The length of each sample is four current cycles. The dataset was labeled as DATASET_E. All samples in the dataset were only used as test samples. Fault features were extracted by using the method in Section 3.2. The well-trained recognition model in Section 4.2 was employed to conduct performance tests. All performance tests were conducted without changing the model parameters of this recognition model. The test results are presented in Table 11.

When the recognition performance tests were conducted with the test samples of these unknown operation conditions, the recognition accuracy of the method slightly decreased. This is because the recognition model has not been trained with samples from these operation conditions. It is a normal phenomenon. In this case, the average recognition accuracy for 1400 test samples under seven different circuit operation parameter conditions is still over 91.9%. It demonstrates that the proposed method has strong adaptability to the changes of circuit operation parameters. It can effectively identify SAFs under different circuit operation parameter conditions.

#### 4.6.2. Effectiveness Tests Under Different SAF Generation Mode Conditions

Series arc fault is a kind of gas discharge phenomenon. Its burning state usually has some fluctuations. This fluctuation will vary signal components of arc current, thereby affecting fault identification results. The arc fault generator used in this work has two kinds of arc generation mode. They are inching mode and vibration mode, respectively. In inching mode, the fluctuation of the burning state is relatively weak. While in vibration mode, the fluctuation is relatively strong. To further evaluate the effectiveness of the proposed method, two groups of SAF experiments under different arc generation mode conditions were conducted by using the experimental platform in Figure 2. The harmonic power source U3 was used as the experimental power supply. The current is 12 A, and the working frequency and PWM carrier frequency of the frequency converter are 50 Hz and 8 kHz, respectively. The sampling frequency of the output current signal is 10 kHz.

For each experiment in Table 12, 100 samples were truncated from measured current signals in normal and fault states. So, there are 200 normal samples and 200 fault samples in the dataset. The length of each sample is four current cycles. The dataset was labeled as DATASET_F. All samples in the dataset were only used as test samples. Fault features were extracted by using the method in Section 3.2. The well-trained recognition model in Section 4.2 was employed to conduct performance tests. The test results are presented in Table 12.

As seen in Table 12, under the same testing conditions, the recognition performance of SAFs generated in vibration mode is better than that in inching mode. It means that SAFs whose burning state fluctuates more obviously are much easier to recognize. The recognition accuracy of SAFs in both kinds of generation mode is higher than 89.5%. So the proposed method has a certain adaptability to the fluctuation of the burning state of SAFs. It is able to identify SAFs with different burning states.

#### 4.6.3. Effectiveness Tests Under Different Fault Location Conditions

In the above-mentioned SAF experiments, all SAFs occurred in the A-phase circuit. The SAFs were successfully identified by analyzing A-phase output current signals. However, in FCCTM circuits, SAFs may occur in any one of A-phase, B-phase or C-phase circuits. To verify the effectiveness of the method when SAFs occur in B-phase or C-phase circuits, recognition performance tests under different fault location conditions were conducted.

Three groups of SAF experiments under different fault location conditions were conducted by using the experimental platform in Figure 2. It was realized by sequentially installing the arc fault generator in A-phase, B-phase and C-phase circuits of the output side of the frequency converter. The harmonic power source U3 was used as the experimental power supply. The current is 12 A, and the working frequency and PWM carrier frequency of the frequency converter are 50 Hz and 8 kHz, respectively. The A-phase output current signal of the frequency converter was collected during each experiment. The sampling frequency was set to 10 kHz. For each experiment in Table 13, 100 samples were truncated from measured current signals in normal and fault states. So, there are 200 normal samples and 200 fault samples in the dataset. The length of each sample is four current cycles. The dataset was labeled as DATASET_G. All samples in the dataset were only used as test samples. Fault features were extracted by using the method in Section 3.2. The well-trained recognition model in Section 4.2 was employed to conduct performance tests. The test results are presented in Table 13.

As shown in Table 13, in FCCTM circuits, when SAFs occur in any one of the phase circuits at the output side of the frequency converter, the faults can be identified with the proposed method by analyzing A-phase output current signals. The recognition accuracy is approximately 90%. Although the occurrence positions of SAFs are different, recognition performance of the method does not vary significantly. Therefore, the proposed method can recognize output-side SAFs occurring in any phase-circuit in FCCTM circuits.

## 5. Conclusions

Since there is no effective recognition method for output-side SAFs in FCCTM circuits, output current signals of frequency converters were first analyzed, and a novel SAF recognition approach was developed. The following conclusions were obtained.

(1) Due to the influence of several factors, such as power quality, working modes of frequency converters and randomness of arc discharge phenomenon, output-side current signals of frequency converters are very complex when output-side SAFs occur. The large number of spikes in current signals will submerge weak SAF features to some extent. Furthermore, the numbers and amplitudes of these spikes have no specific variation pattern. So it is a challenge to precisely recognize output-side SAFs in FCCTM circuits via output-side current signals.

(2) A lot of high-frequency components will appear in current signals even if the circuit operates normally, low-frequency characteristics of current signals should be applied to recognize output-side SAFs. An AR model was established by using the first three modal components of EWT decomposition results of current signals. AR model parameters and the energy ratios of those modal components can be applied to construct fault features.

(3) The presented recognition scheme based on EWT and SVM can successfully recognize output-side SAFs in FCCTM circuits. Compared with existing methods, the proposed method has higher recognition accuracy and shorter runtime. Furthermore, it has certain anti-interference ability and adaptability to different circuit operation conditions.

In this work, the effectiveness of the proposed method was verified only by using the experimental data of a specific FCCTM circuit in Figure 2. Due to the limitations of the experimental platform, recognition performance tests under the conditions of different frequency converter topologies, motor types and technical parameters have not yet been conducted. Considering numerous complicated factors in engineering applications, more performance tests are needed in the future to assess the adaptability of this method to more FCCTM circuit configurations, as well as to special application scenarios such as frequency converter ride-through fault, sensor temperature drift and multiple fault cases. Furthermore, deploying the method into target embedded platforms such as Raspberry Pi, Jetson nano, and DSP, and then developing industrial microcomputer-based arc fault protection devices suitable for FCCTM circuits based on the existing AFCI standards [28,39] will be also one of the main tasks in the future.

## Figures and Tables

**Table 1:** Experimental conditions.

Group No.	Power Source	Experimental State
1–6	U1–U6	Normal
7–12	U1–U6	Fault

**Table 2:** Harmonic parameters of U3-U6.

Harmonic Number	Harmonic Contents and Phase Angle			
	U3	U4	U5	U6
3rd	1.1%/0°	2.2%/0°	4.9%/0°	3.0%/180°
5th	2.8%/0°	5.6%/0°	1.6%/0°	2.75%/0°
7th	1.4%/0°	2.8%/0°	2.7%/0°	2.4%/180°
9th	2.3%/0°	4.6%/0°	0.0%/0°	2.0%/0°
11th	1.5%/0°	3.0%/0°	1.4%/0°	1.4%/180°
13th	0.0%/0°	0.0%/0°	0.0%/0°	0.8%/0°
15th	0.0%/0°	1.4%/0°	2.0%/0°	0.0%/0°
17th	0.0%/0°	0.0%/0°	1.1%/0°	0.0%/0°
21st	0.0%/0°	1.0%/0°	0.0%/0°	0.0%/0°

**Table 3:** Energy ratios of different modal components.

Modal Component	Energy Ratio/%	
	In Normal State	In Fault State
f1	11.7	3.9
f2	85.0	30.2
f3	0.3	61.3
f4	0.7	0.4
f5	0.4	1.1
f6	0.3	0.6
f7	0.1	0.2
f8	1.4	2.1

**Table 4:** Recognition results of SAFs under different power source conditions.

Group No.	Power Source	Accuracy /%	Precision /%	Recall /%	F1-Score	FPR /%	FNR /%
A1	U1	99.0	100.0	98.0	0.99	0.0	2.0
A2	U2	95.5	96.0	95.0	0.96	4.0	5.0
A3	U3	97.5	99.0	96.0	0.98	1.0	4.0
A4	U4	98.0	99.0	97.0	0.98	1.0	3.0
A5	U5	97.5	97.0	98.0	0.98	3.0	2.0
A6	U6	95.0	97.9	92.0	0.95	2.0	8.0
Mean	–	97.1	98.1	96.0	0.97	1.8	4.0

**Table 5:** Recognition results under different *K* and *M* conditions.

*K*	*M*	Accuracy/%	*K*	*M*	Accuracy/%
4	3	95.2	8	1	87.8
6	3	94.3	8	2	95.8
8	3	97.1	8	4	96.3
10	3	96.3	8	5	95.7
12	3	96.0	8	6	94.4

**Table 6:** Test results under different sampling frequency and sample length conditions.

Group No.	Sampling Frequency /kHz	Sample Length /Number of Current Cycles	Accuracy /%	Precision /%	Recall /%	F1-Score
B11	10	1	79.3	83.7	72.7	0.78
B12	10	2	89.1	91.3	86.3	0.89
B13	10	3	92.2	92.3	92.0	0.92
B14	10	4	97.1	98.1	96.0	0.97
B15	10	5	95.9	96.8	95.0	0.96
B16	10	6	96.7	97.5	95.8	0.97
B17	10	7	97.0	98.0	96.0	0.97
B21	10	4	97.1	98.1	96.0	0.97
B22	5.0	4	91.5	94.4	88.2	0.91
B23	2.5	4	85.8	85.2	86.5	0.86

**Table 7:** Recognition results and average runtime by using different signal decomposition methods.

Group No.	Method	Accuracy /%	Precision /%	Recall /%	F1-Score	FPR /%	FNR /%	Runtime /ms
C11	EMD	63.8	63.4	65.0	0.64	37.5	35.0	1.93
C12	WPT	61.3	62.4	56.7	0.59	34.2	43.3	5.68
C13	VMD	68.6	71.0	62.8	0.67	25.7	37.2	2812
C14	EWT	97.1	98.1	96.0	0.97	1.8	4.0	1.00

**Table 8:** Recognition results by using different feature extraction methods.

Group No.	Method	Accuracy /%	Precision /%	Recall /%	F1-Score	FPR /%	FNR /%	Runtime /ms
C21	Method 1 [35]	59.6	64.1	43.7	0.52	24.5	56.3	2.1
C22	Method 2 [7]	88.4	89.4	87.2	0.88	10.3	12.8	0.73
C23	Method 3 [23]	60.1	63.5	47.5	0.54	27.3	52.5	13.3
C24	Method 4 [30]	89.5	92.3	86.2	0.89	7.2	13.8	1.2
C25	Method 5 [36]	80.9	84.3	76.0	0.80	14.2	24.0	0.1
C26	Method 6 [37]	74.5	77.9	68.3	0.73	19.3	31.7	1.0
C27	Method 7 [38]	92.4	94.9	89.7	0.92	4.8	10.3	249.8
C28	Method 8	93.0	92.9	93.2	0.93	7.2	6.8	1.23
C29	Proposed	97.1	98.1	96.0	0.97	1.8	4.0	1.4

**Table 9:** Test results under Gaussian white noise conditions.

Group No.	SNR /dB	Accuracy /%	Precision /%	Recall /%	F1-Score	FPR /%	FNR /%
D11	30	96.5	97.3	95.7	0.97	2.7	4.3
D12	25	95.2	93.3	97.3	0.95	7.0	2.7
D13	20	68.6	67.4	72.0	0.70	34.8	28.0

**Table 10:** Test results under pink noise conditions.

Group No.	SNR /dB	Accuracy /%	Precision /%	Recall /%	F1-Score	FPR /%	FNR /%
D21	40	96.0	97.1	94.8	0.96	2.8	5.2
D22	35	96.2	96.3	96.0	0.96	3.7	4.0
D23	30	68.3	65.3	78.2	0.71	41.5	21.8

**Table 11:** Test results under different circuit operation parameter conditions.

Group No.	Working Current /A	Working Frequency /Hz	Carrier Frequency /kHz	Accuracy /%	Precision /%	Recall /%	F1-Score	FPR /%	FNR /%
E1	15	30	8	92.0	94.7	89.0	0.92	5.0	11.0
E2	15	40	8	89.5	94.4	84.0	0.89	5.0	16.0
E3	15	30	3	97.0	95.2	99.0	0.97	5.0	1.0
E4	15	50	3	95.0	96.9	93.0	0.95	3.0	7.0
E5	12	50	8	89.0	93.3	84.0	0.88	6.0	16.0
E6	14	50	8	88.0	88.8	87.0	0.88	11.0	13.0
E7	12–16	50	8	92.5	92.1	93.0	0.93	8.0	7.0
Mean	–	–	–	91.9	93.6	89.9	0.92	6.1	10.1

**Table 12:** Test results under different SAF generation mode conditions.

Group No.	Power Source	SAF Generation Mode	Accuracy /%	Precision /%	Recall /%	F1-Score	FPR /%	FNR /%
F1	U3	Inching	89.5	89.9	89.0	0.89	10.0	11.0
F2	U3	Vibration	92.0	89.6	95.0	0.92	11.0	5.0
Mean	–	–	90.8	89.8	92.0	0.91	10.5	8.0

**Table 13:** Test results under different fault location conditions.

Group No.	Power Source	Fault Location	Accuracy /%	Precision /%	Recall /%	F1-Score	FPR /%	FNR /%
G1	U3	A-phase	95.0	91.7	99.0	0.95	9.0	1.0
G2	U3	B-phase	89.5	89.9	89.0	0.89	10.0	11.0
G3	U3	C-phase	89.0	85.5	94.0	0.90	16.0	6.0
Mean	–	–	91.2	89.0	94.0	0.90	11.7	6.0

## Data Availability

The data presented in this work are available on reasonable request from the corresponding author.

## Abbreviations

AFCI	Arc Fault Circuit Interrupter
AFG	Arc Fault Generator
AR model	Autoregressive Model
CNN	Convolutional Neural Network
DAC	Data Acquisition Card
EMD	Empirical Mode Decomposition
EWT	Empirical Wavelet Transform
FCCTM	Frequency Converter-Controlled Three-phase Motor
FNR	False Negative Rate
FPR	False Positive Rate
GS	Grid Search
GS-PSO	Grid Search with Particle Swarm Optimization
PSO	Particle Swarm Optimization
SAF	Series Arc Fault
SNR	Signal-to-Noise Ratio
SVD	Singular Value Decomposition
SVM	Support Vector Machine
VMD	Variational Mode Decomposition
WPT	Wavelet Packet Transform
WT	Wavelet Transform
